# Supplementary material for: Non-Ionic Osmotic Stress Induces the Biosynthesis of Nodulation Factors and Affects Other Symbiotic Traits in Sinorhizobium fredii HH103
Source: Biology (Basel). 2023 Jan 18;12(2):148. doi: 10.3390/biology12020148 (PMC9952627; doi:10.3390/biology12020148)
Supplement: Supplementary file 1 [file biology-12-00148-s001.zip › FuentesRomeroetalBiology-2146600SuppRev2.pdf]

Non-Ionic Osmotic Stress Induces the Biosynthesis of Nodulation Factors and Affects Other Symbiotic Traits in *Sinorhizobium fredii* HH103

Francisco Fuentes-Romero[1], Isamar Moyano-Bravo[1], Paula Ayala-García[1], Miguel Ángel Rodríguez-Carvajal[2], Francisco Pérez-Montaño[1], Sebastián Acosta-Jurado[3], Francisco Javier Ollero[1], José María Vinardell[1,*]

List of Supplementary Material

Figure S1. β-galactosidase activity of *S. fredii* HH103 carrying plasmid pMP240 upon growth in YM supplemented with different mannitol concentration (from 55 mM to 1 M).

Figure S2. Growth curves of *S. fredii* HH103 in different media supplemented or not with 400 mM mannitol. (**A**) TY medium; (**B**) YM medium; (**C**) Minimal medium (MM); (**D**) Bromfield medium (BF).

Figure S3. LPS (**A**) and KPS (**B**) electrophoretic profiles of *S. fredii* HH103 grown in TY medium in the absence or presence of 400 mM mannitol.

Table S1. Fold-changes, as determined by RNAseq, of the *S. fredii* HH103 *nodABC* genes after 16-h growth in the presence of 400 mM mannitol.

Supplementary Dataset S1. Complete list of Nod factors produced by *Sinorhizobium fredii* HH103 in YM medium in the absence (HH-) or the presence of 3.7 μM genistein (HH+) or 400 mM mannitol (HH mannitol).

Supplementary Dataset S2. *S. fredii* HH103 DEGs upon growth in 400 mM mannitol ordered by fold-change.

Supplementary Dataset S3. List of *S. fredii* HH103 induced genes in the presence of 400 mM mannitol, ordered by replicon.

Supplementary Dataset S4. List of the different AHLs detected as produced by *S. fredii* HH103 grown in the absence (269) or presence (269_Manitol) of 400 mM mannitol.

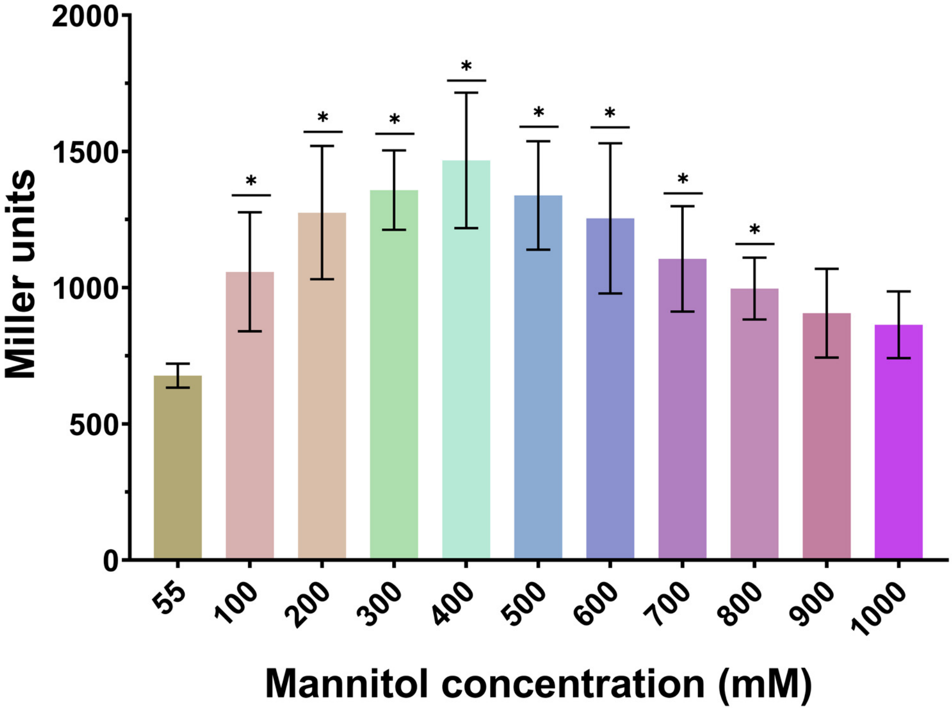

Figure S1. β-galactosidase activity of *S. fredii* HH103 carrying plasmid pMP240 upon growth in YM supplemented with different mannitol concentration (from 55 mM to 1 M). Asterisks (*) indicate significant differences with the corresponding control sample using the non-parametric test of Mann–Whitney, $\alpha = 5\%$.

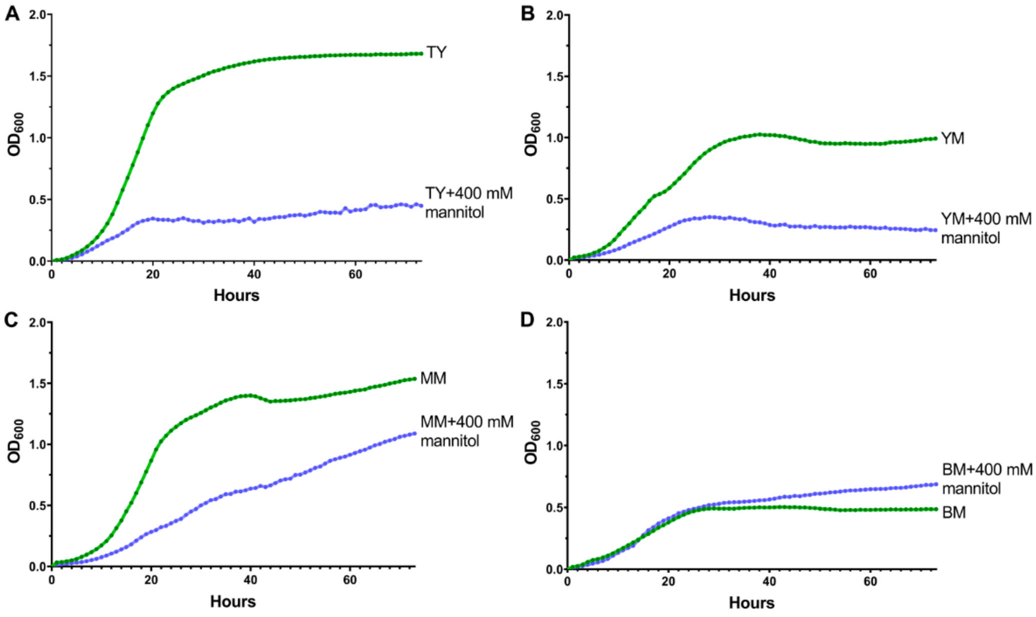

Figure S2. Growth curves of *S. fredii* HH103 in different media supplemented or not with 400 mM mannitol. (**A**) TY medium; (**B**) YM medium; (**C**) Minimal medium (MM); (**D**) Bromfield medium (BF).

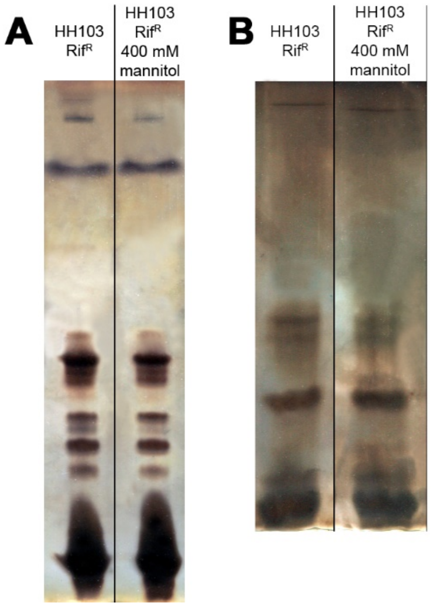

Figure S3. LPS (**A**) and KPS (**B**) electrophoretic profiles of *S. fredii* HH103 grown in TY medium in the absence or presence of 400 mM mannitol.

Table S1. Fold-changes, as determined by RNAseq, of the *S. fredii* HH103 *nodABC* genes after 16-h growth in the presence of 400 mM mannitol.

Gene_name	GENEID	log2BaseMean	log2Ratio	STDERR_log2Ratio	pvalue	padjust	FoldChange
nodA	psfHH103d_126	9.408819572	1.0628198	0.615502891	0.08421252	0.20073394	2.08901058
nodB	psfHH103d_127	9.451582027	0.90654382	0.442138801	0.04032929	0.11817993	1.87454936
nodC	psfHH103d_128	9.235802154	1.4530014	0.448751746	0.00120424	0.00866408	2.73777029
