# Supplementary figures and images for: Non-Ionic Osmotic Stress Induces the Biosynthesis of Nodulation Factors and Affects Other Symbiotic Traits in Sinorhizobium fredii HH103
Source: Biology (Basel). 2023 Jan 18;12(2):148. doi: 10.3390/biology12020148 (PMC9952627; doi:10.3390/biology12020148)

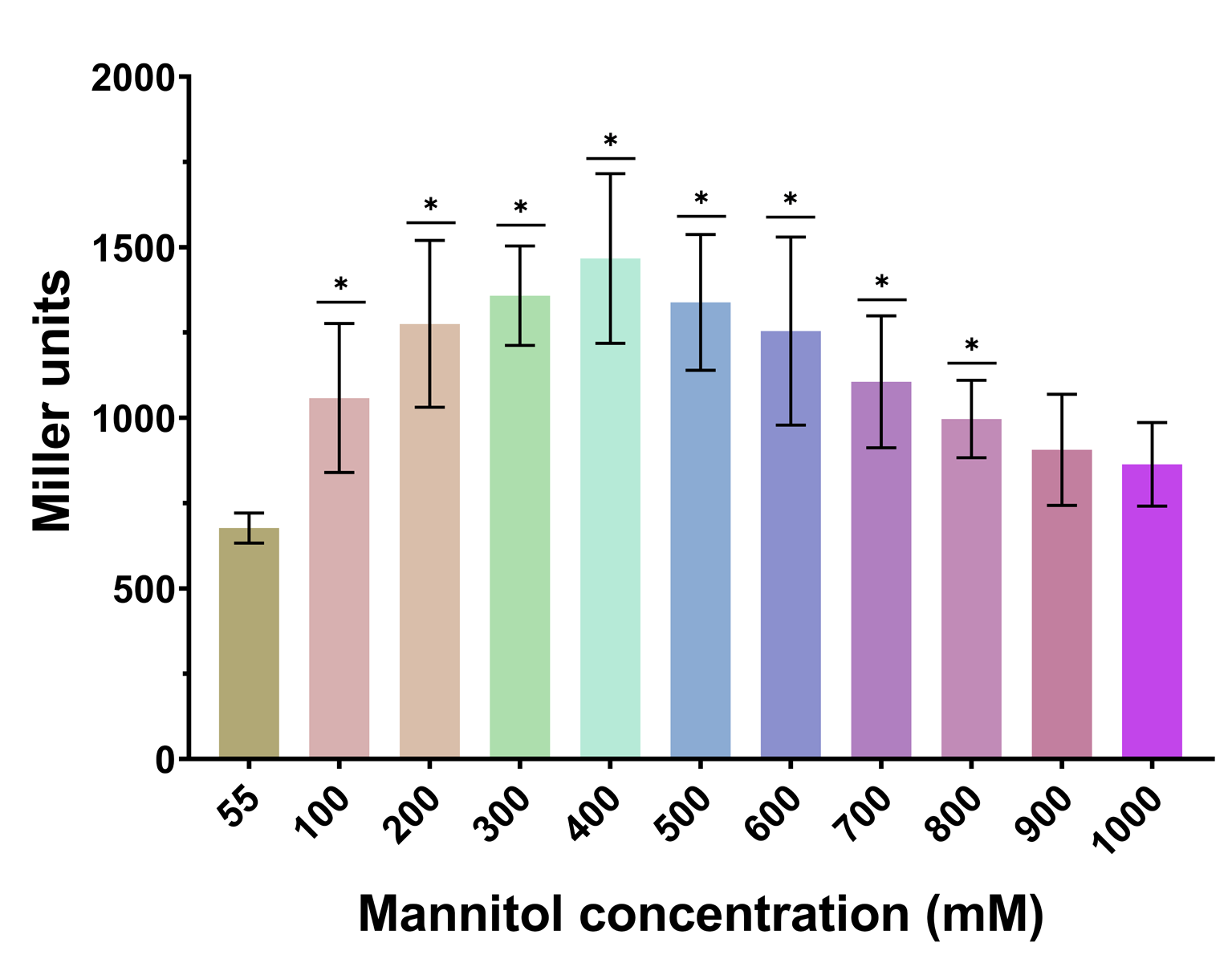

Supplement: Supplementary file 1 [file biology-12-00148-s001.zip › Figure S1_rev.tif]

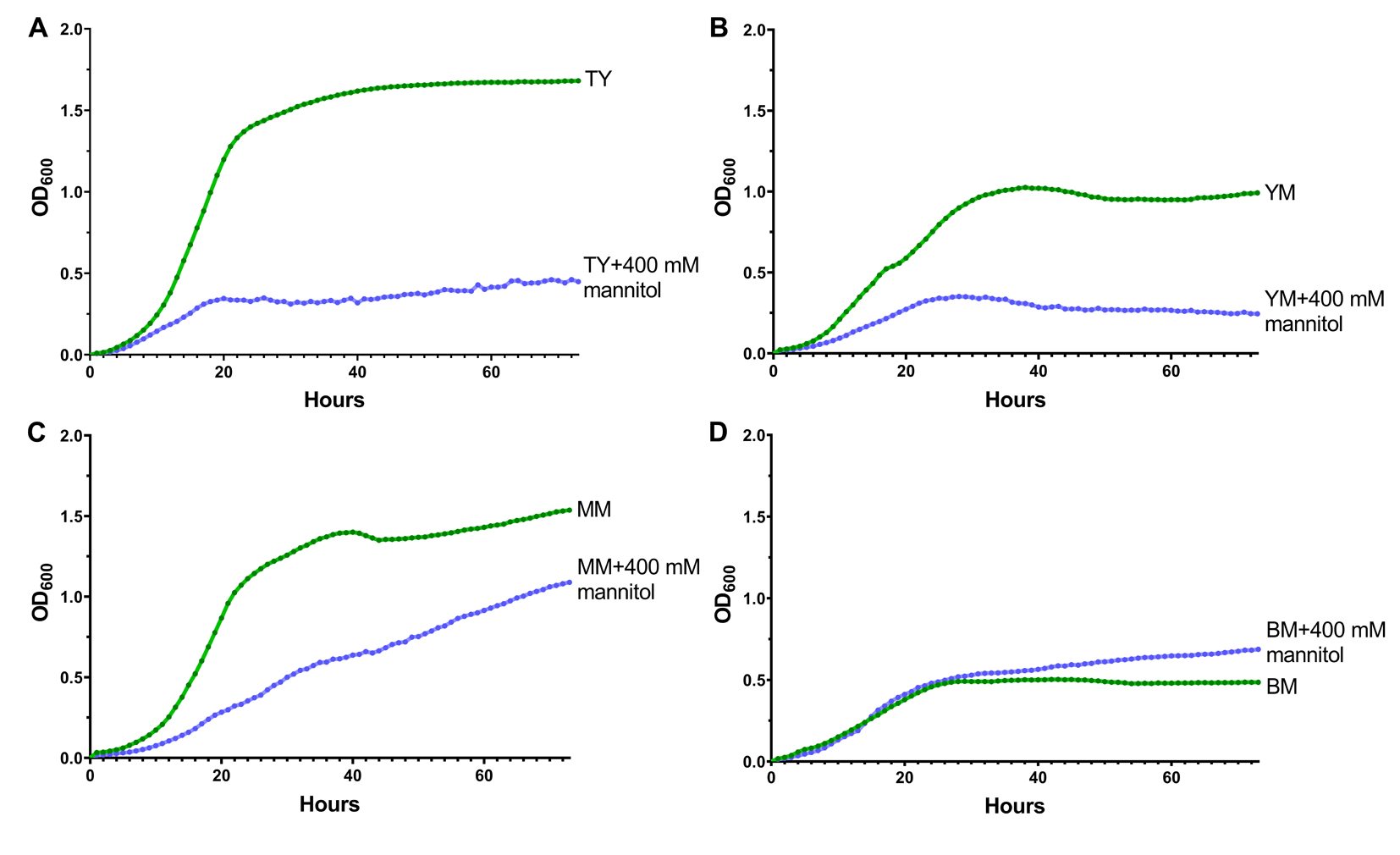

Supplement: Supplementary file 1 [file biology-12-00148-s001.zip › Figure S2.tif]

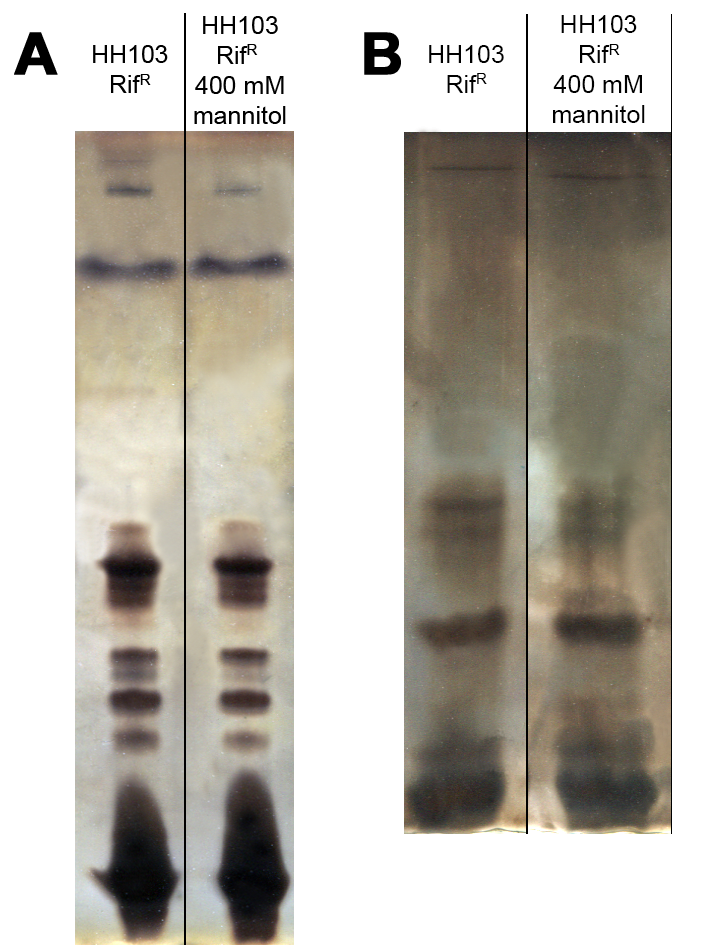

Supplement: Supplementary file 1 [file biology-12-00148-s001.zip › Figure S3.tif]
